# Interaction of left ventricular size with the outcome of cardiac resynchronization therapy in Japanese patients

**DOI:** 10.1002/clc.24267

**Published:** 2024-04-15

**Authors:** Ryo Ito, Yusuke Kondo, Masahiro Nakano, Takatsugu Kajiyama, Miyo Nakano, Mari Kitagawa, Masafumi Sugawara, Toshinori Chiba, Yoshio Kobayashi

**Affiliations:** ^1^ Department of Cardiovascular Medicine Chiba University Graduate School of Medicine Chiba Japan; ^2^ Department of Advanced Cardiorhythm Therapeutics Chiba University Graduate School of Medicine Chiba Japan

**Keywords:** cardiac resynchronization therapy, left ventricle size, LV end‐diastolic volume, QRS duration

## Abstract

**Background:**

We analyzed the influence of the QRS duration (QRSd) to LV end‐diastolic volume (LVEDV) ratio on cardiac resynchronization therapy (CRT) outcomes in heart failure patients classified as III/IV per the New York Heart Association (NYHA) and with small body size.

**Hypothesis:**

We proposed the hypothesis that the QRSd/LV size ratio is a better index of the CRT substrate.

**Methods:**

We enrolled 114 patients with advanced heart failure (NYHA class III/IV, and LV ejection fraction >35%) who received a CRT device, including those with left bundle branch block (LBBB) and QRSd ≥120 milliseconds (*n* = 60), non‐LBBB and QRSd ≥150 milliseconds (*n* = 30) and non‐LBBB and QRSd of 120−149 milliseconds (*n* = 24).

**Results:**

Over a mean follow‐up period of 65 ± 58 months, the incidence of the primary endpoint, a composite of all‐cause death and hospitalization for heart failure, showed no significant intergroup difference (43.3% vs. 50.0% vs. 37.5%, respectively, *p* = .72). Similarly, among 104 patients with QRSd/LVEDV ≥ 0.67 (*n* = 54) and QRSd/LVEDV < 0.67 (*n* = 52), no significant differences were observed in the incidence of the primary endpoint (35.1% vs. 51.9%, *p* = .49). Nevertheless, patients with QRSd/LVEDV ≥ 0.67 showed better survival than those with QRSd/LVEDV < 0.67 (14.8% vs. 34.6%, *p* = .0024).

**Conclusion:**

Advanced HF patients with a higher QRSd/LVEDV ratio showed better survival in this small‐body–size population. Thus, the risk is concentrated among those with a larger QRSd, and patients with a relatively smaller left ventricular size appeared to benefit from CRT.

## Graphical abstract

Advanced HF patients with a higher QRS duration (QRSd)/LV end‐diastolic volume (LVEDV) ratio showed better survival in this small‐body–size population. Thus, the QRSd/LVEDV might serve as a relatively simple index to improve patient selection for cardiac resynchronization therapy in patients with mid‐wide QRSd.

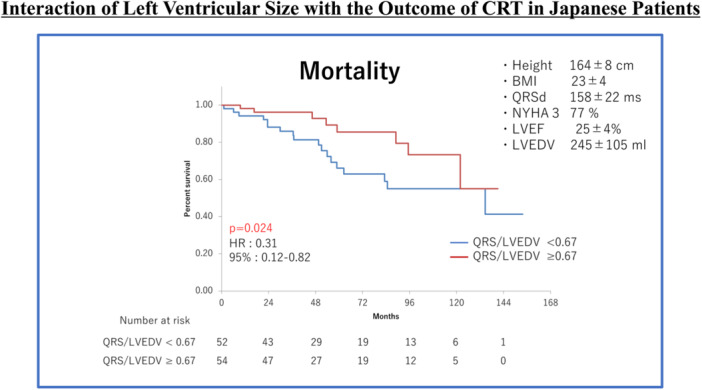

## 1 INTRODUCTION

Cardiac resynchronization therapy (CRT) is an effective therapeutic modality for patients with heart failure and reduced left ventricular ejection fraction (HFrEF). CRT increases the coordination and the efficiency of contraction of the heart, improves left ventricle (LV) systolic function and quality of life, and reduces heart failure symptoms, hospitalizations, and mortality.^1^, ^2^, ^3^ Assessment of QRS duration (QRSd) is a key selection criterion for CRT, which shows a spectrum of responses. The indications reflect the greatest probability for successful therapy with QRSd >150 milliseconds, less (or no) effect for QRSd <150 milliseconds, and futility (or possible harm) with QRSd <130 milliseconds.^4^

The Echocardiography Guided Cardiac Resynchronization Therapy (EchoCRT) trial, which evaluated the effects of CRT in patients with QRSd <130 milliseconds and LV dilatation, suggested that CRT shows contrasting effects among patients with heart failure with QRSd <130 milliseconds according to LV size: worsening outcomes in patients with larger LV, but beneficial effects in those with a smaller LV.^5^, ^6^ In addition, the QRSd/LV size ratio may be a better index of the CRT substrate, and patients with a high index score (i.e., a relatively large QRSd and small LV size) show a higher probability of a beneficial CRT response than those with a low index score (smaller QRSd and large LV size), whose condition could worsen with treatment. Therefore, we aimed to analyze the influence of the QRS/LV end‐diastolic volume (LVEDV) ratio on the clinical outcome of CRT in HFrEF patients with a small body size and New York Heart Association (NYHA) classification III/IV.

## 2 METHODS

### 2.1 Study design and population

The database of the cardiac implantable electronic device (CIED) clinic of Chiba University Hospital, Japan, was retrospectively analyzed. Patients who received CRT with a defibrillator device between January 2008 and December 2018 were enrolled in this study. Patients who had persistent atrial fibrillation (AF) were excluded. Patients with mild heart failure symptoms (NYHA II) or LVEF > 35% at implantation of the CRT defibrillator device (CRTD) were also excluded. We enrolled patients with sinus rhythm, NYHA III/IV, and LVEF ≤ 35% and divided them into the following groups according to the CRT indication of the Japanese Circulation Society (JCS)/Japanese Heart Rhythm Society (JHRS) Guideline on Non‐Pharmacotherapy of Cardiac Arrhythmias: (i) left bundle branch block (LBBB) patients with QRSd ≥120 milliseconds; (ii) non‐LBBB patients with QRSd ≥150 milliseconds; and (iii) non‐LBBB patients with QRSd of 120−149 milliseconds.^7^ These groups corresponded to CRT indication classes I, IIa, and IIb, and the groups were defined as class I, IIa, and IIb groups, respectively. We compared the outcomes of CRT therapy in the three groups and evaluated the influence of QRSd/LVEDV on the clinical outcome. QRSd was determined from a standard 12‐lead ECG recorded at 25 mm/s before implantation. LV size was derived from the preimplantation biplane LVEDV. Informed consent was obtained using the opt‐out method with a poster which was approved by the Ethical Committee of Chiba University. This study complied with the Declaration of Helsinki and was approved by the Ethics Committees of all involved institutions.

### 2.2 Treatment

Drug therapy with agents such as β‐blockers may improve cardiac function and generate reverse remodeling of the LV in patients with HF. Therefore, the indications for treatment should be considered after sufficient pharmacotherapy. CRT, in particular, is not indicated within 3 months after revascularization and within 3 months after the introduction of new pharmacotherapy for heart failure, except for special reasons. Meanwhile, the dose of β‐blockers can be increased after CRT induction in some patients; thus, CRT may be indicated in situations in which maximization of the dose cannot be achieved. The CRTD was programmed at the physician's discretion.

### 2.3 Endpoints

The primary endpoint was a composite of all‐cause mortality and hospitalization for heart failure. The secondary endpoint was appropriate defibrillator therapy and stroke events after CRTD implantation. Stroke was defined as a sudden onset of a focal neurological deficit lasting >24 hours and further categorized as ischemic or hemorrhagic. These events were evaluated throughout the follow‐up period from the date of CRTD implantation. Follow‐up data after discharge were obtained by a review of medical records or a telephone interview with the patient or his/her family members.

### 2.4 Statistical analysis

Statistical analysis was performed using the EXCEL software package version 3.1. Continuous variables were expressed as mean ± standard deviation when normally distributed and as median and interquartile range when non‐normally distributed. Categorical data are presented as absolute numbers and percentages. Continuous variables were compared using Student's *t*‐test or the Mann–Whitney *U* test, as appropriate. Categorical variables were compared using the *χ* ^2^ test or Fisher's exact test. The cumulative event‐free survival rates were calculated using the Kaplan–Meier method and compared using the log‐rank test. A Cox proportional‐hazards model was used to estimate unadjusted and adjusted hazard ratios with the corresponding 95% confidence intervals (CIs). A *p*‐value < .05 was considered statistically significant.

## 3 RESULTS

**Figure 1:**
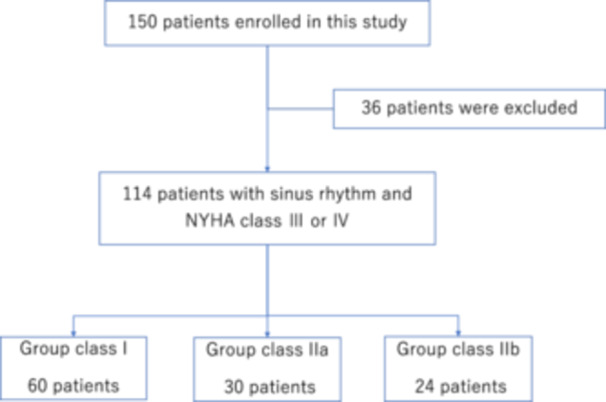
Flowchart of the study population. NYHA, New York Heart Association functional classification.

**Figure 2:**
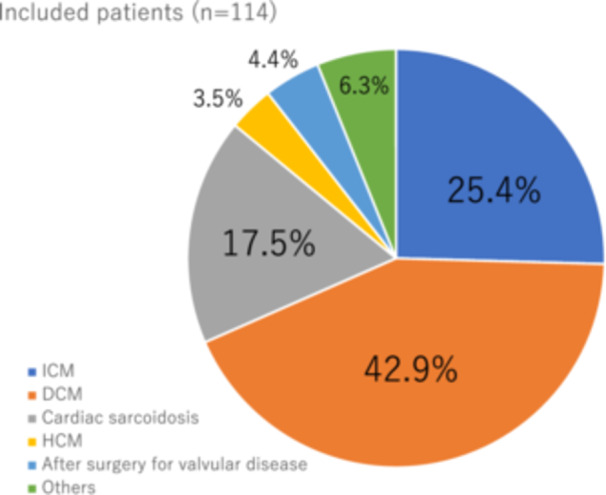
The underlying disease of included patients. DCM, dilated cardiomyopathy; HCM, hypertrophic cardiomyopathy; ICM, ischemic cardiomyopathy.

We enrolled 150 consecutive patients who were followed‐up at our CIED clinic. Figure 1 shows the flow diagram of this study. Thirty‐six patients with persistent AF, LVEF > 35%, or NYHA II were excluded. Thus, 114 patients were included in this study (age, 70 ± 16 years; male sex, 64%; body mass index, 23 ± 4 kg/m^2^). Figure 2 demonstrates the underlying diseases for CRT. All patients underwent a triple‐chamber CRTD implantation based on a class I or II indication according to JCS/JHRS guidelines.^7^ Over a mean follow‐up period of 65 ± 58 months, the primary endpoints occurred in 63 (10.38% per year) of the 114 patients: all‐cause death and hospitalization for heart failure occurred in 30 (5.22% per year) and 33 (6.85% per year) patients, respectively. The secondary endpoints of appropriate implantable cardioverter defibrillator therapy and stroke occurred in 16 (3.09% per year) and 12 (2.13% per year) patients, respectively.

### 3.1 Classification based on the JCS guidelines and outcomes

The 114 patients were divided into groups corresponding to class I, class IIa, and class IIb indications. The baseline characteristics of each group are summarized in Table 1A. The period from the first onset of HF to CRTD implantation was significantly the shortest in the class IIa group (1139 ± 1711 vs. 500 ± 869 vs. 1351 ± 2184 days, respectively, *p* = .00122).

**Table 1:** Baseline characteristics of patients with (A) class I, class IIa, and class IIb and (B) QRSd/LVEDV ≥ 0.67 and QRSd/LVEDV < 0.67.

(A)					
	Total (*n* = 114)	Class I (*n* = 60)	Class IIa (*n* = 30)	Class IIb (*n* = 24)	*p* Value
Follow‐up periods (days)	1839 ± 1139	1919 ± 1202	1860 ± 1202	1610 ± 882	.25
Age (year)	64.0 ± 9.2	64.9 ± 9.7	64.0 ± 8.4	62.0 ± 8.9	.21
Female, *n* (%)	34 (29.8)	15 (25.0)	10 (33.3)	9 (37.5)	.12
Height (cm)	163.9 ± 8.0	165.0 ± 7.8	162.1 ± 8.1	163.2 ± 8.1	.090
BMI	23.1 ± 3.8	23.1 ± 3.9	22.6 ± 3.4	23.5 ± 4.1	.36
QRS duration (ms)	158.1 ± 22.1	158.9 ± 22.7	172.7 ± 16.8	137.8 ± 5.5	<.001
Primary prevention, *n* (%)	88 (77.1)	53 (88.3)	20 (66.6)	15 (62.5)	.0032
NYHA class III, *n* (%)	88 (77.1)	49 (81.6)	20 (66.6)	19 (79.1)	.056
LVEF (%)	25.2 ± 7.9	23.7 ± 7.0	25.6 ± 7.9	27.3 ± 7.8	.046
LVDd (mm)	67.8 ± 10.9	70.4 ± 9.8	65.2 ± 10.7	64.7 ± 12.3	.030
LVEDV (mL)	244.7 ± 105.0	267.2 ± 105.4	226.3 ± 90.5	212.7 ± 112.9	.048
QRS/LVEDV	0.785 ± 0.415	0.685 ± 0.251	0.892 ± 0.367	0.895 ± 0.681	.050
ICM, *n* (%)	29 (25.4)	16 (26.6)	9 (30.0)	4 (16.6)	.13
DCM, *n* (%)	49 (42.9)	36 (60.0)	6 (20.0)	7 (29.1)	<.001
CHA2DS2‐VASc score	2.94 ± 1.33	3.05 ± 1.37	3.00 ± 1.46	2.62 ± 1.05	.30
HAS‐BLED score	1.00 ± 0.86	1.13 ± 0.87	0.96 ± 0.76	0.70 ± 0.90	.0495
Stroke, *n* (%)	12 (10.5)	5 (8.3)	4 (13.3)	3 (12.5)	.46
BNP (pg/mL)	690.1 ± 901.0	715.0 ± 975.5	694.4 ± 848.1	622.4 ± 796.2	.68
Beta‐blockers, *n* (%)	108 (94.7)	58 (96.6)	27 (90.0)	23 (95.8)	.096
ACEI or ARB, *n* (%)	100 (87.7)	53 (88.3)	28 (93.3)	19 (79.1)	.061
Aldosterone antagonists, *n* (%)	75 (65.7)	39 (65.0)	20 (66.6)	16 (66.6)	.44
Diuretics, *n* (%)	87 (76.3)	47 (78.3)	24 (80.0)	16 (66.6)	.13
Cardiotonic, *n* (%)	19 (16.6)	9 (15.0)	7 (23.3)	3 (12.5)	.15
Amiodarone, *n* (%)	43 (37.7)	22 (36.6)	10 (33.3)	11 (45.8)	.17
Antiplatelet agents, *n* (%)	35 (30.7)	18 (30.0)	12 (40.0)	5 (20.8)	.040
Days from the first onset of HF to CRTD implant (days)	1138.5 ± 1711.7	1372.6 ± 1760.6	500.1 ± 869.4	1351.3 ± 2183.6	.012

(B)		
	QRS/LVEDV ≥0.67 (*n* = 54)	QRS/LVEDV <0.67 (*n* = 52)	*p* Value
Follow‐up periods (days)	1852 ± 1124	1872 ± 1188	.92
Age (year)	66.3 ± 8.1	62.0 ± 9.9	.015
Female, *n* (%)	21 (38.8)	11 (21.1)	.023
Height (cm)	161.0 ± 7.0	166.2 ± 7.8	<.001
BMI	23.0 ± 3.1	23.1 ± 4.4	.92
QRS duration (ms)	156.5 ± 20.1	161.8 ± 24.0	.22
Primary prevention, *n* (%)	42 (77.7)	40 (76.9)	.46
NYHA class III, *n* (%)	45 (83.3%)	38 (73.0%)	.10
LVEF (%)	29.3 ± 7.2	20.9 ± 5.7	<.001
LVDd (mm)	60.9 ± 7.7	76.1 ± 7.7	<.001
LVEDV (mL)	161.9 ± 42.9	330.7 ± 77.3	<.001
QRS/LVEDV	1.051 ± 0.427	0.510 ± 0.116	<.001
ICM, *n* (%)	14 (25.9)	12 (23.0)	.36
PAF, *n* (%)	10 (18.5)	6 (11.5)	.16
CHA_2_DS_2_‐VASc score	3.37 ± 1.35	2.53 ± 1.17	.0010
HAS‐BLED score	1.11 ± 0.86	0.86 ± 0.79	.13
Stroke, *n* (%)	4 (7.4)	7 (13.4)	.15
BNP (pg/mL)	424.5 ± 465.3	1007.4 ± 1160.9	<.001
Beta‐blockers, *n* (%)	52 (96.2)	48 (92.3)	.19
ACEI or ARB, *n* (%)	50 (92.5)	45 (86.5)	.15
Aldosterone antagonists, *n* (%)	32 (59.2)	38 (73.0)	.067
Diuretics, *n* (%)	38 (70.3)	47 (90.3)	.0049
Cardiotonic, *n* (%)	4 (7.4)	13 (25.0)	.0068
Amiodarone, *n* (%)	17 (31.4)	21 (40.3)	.17
Oral anticoagulant agents, *n* (%)	22 (40.7)	25 (48.0)	.22
Days from the first onset of HF to CRTD implant (days)	677 ± 1073	1618 ± 2054	.0037

Abbreviations: ACEI, angiotensin converting enzyme inhibitor; ARB, angiotensin II receptor blocker; BMI, body mass index; BNP, brain natriuretic peptide; CRTD, CRT defibrillator device; DCM, dilated cardiomyopathy; ICM, ischemic cardiomyopathy; LVDd, left ventricular end‐diastolic diameter; LVEDV, left ventricular end‐diastolic volume; LVEF, left ventricular ejection fraction; NYHA, New York Heart Association functional classification; QRSd, QRS duration.

**Figure 3:**
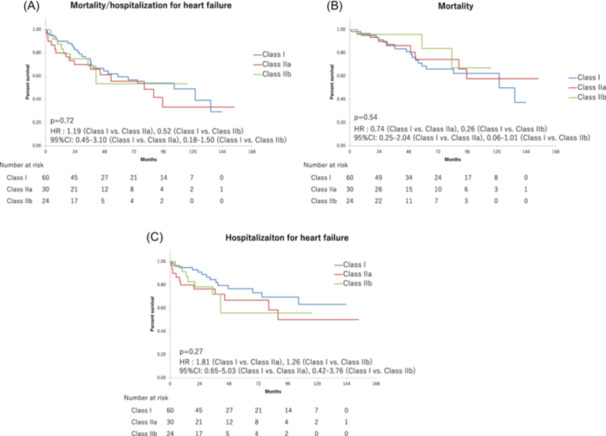
Kaplan−Meier curves for the probability of event‐free survival (class I vs. class IIa vs. class IIb). Composite of all‐cause mortality and hospitalization for heart failure (A). All‐cause mortality (B) hospitalization for heart failure (C).

The incidence of the primary endpoint did not differ significantly among the groups (43.3% vs. 50.0% vs. 37.5%, respectively, *p* = .72) (Figure 3A). There were no significant differences among the groups in the incidence of all‐cause death and hospitalization for heart failure (31.6% vs. 26.6% vs. 12.5%, respectively, *p* = .54; Figures 3B,C), and there were no significant differences in the incidence of the secondary endpoints of appropriate defibrillator therapy (8.3% vs. 23.3% vs. 16.6%, respectively, *p* = .06; Figure SA).

### 3.2 QRSd/LVEDV ratio and outcomes

The mean QRSd and LVEDV were 158 ± 22 milliseconds and 244 ± 105 mL, respectively. The mean QRSd/LVEDV was 0.78 ± 0.41. Receiver operating characteristic (ROC) curve analysis was performed, and the area under the curve (AUC) was calculated to determine the best QRSd/LVEDV for predicting the risk of embolic stroke onset. ROC curve analysis confirmed that 0.67 was the best cut‐off point for all‐cause death (AUC = 0.62) (Figure SB). Among the 114 patients, 10 patients were excluded because of a lack of detailed echocardiography data. The remaining 104 patients were divided into groups with QRSd/LVEDV ≥ 0.67 (*n* = 54, 51.9%) and QRSd/LVEDV < 0.67 (*n* = 52, 48.1%). The baseline characteristics of each group are summarized in Table 1B. Patients with QRSd/LVEDV ≥ 0.67 were older and smaller than patients with QRSd/LVEDV < 0.67 (66.3 ± 8.1 vs. 62.0 ± 9.9 years; *p* = .015; 161.0 ± 7.0 vs. 166.2 ± 7.8 cm, *p* < .001). The proportion of females in the group with QRSd/LVEDV ≥ 0.67 was greater than that in the group with QRSd/LVEDV < 0.67 (38.8% vs. 21.1%, *p* = .023). Furthermore, patients with QRSd/LVEDV ≥ 0.67 had a smaller LV volume and better LVEF than patients with QRSd/LVEDV < 0.67.

**Figure 4:**
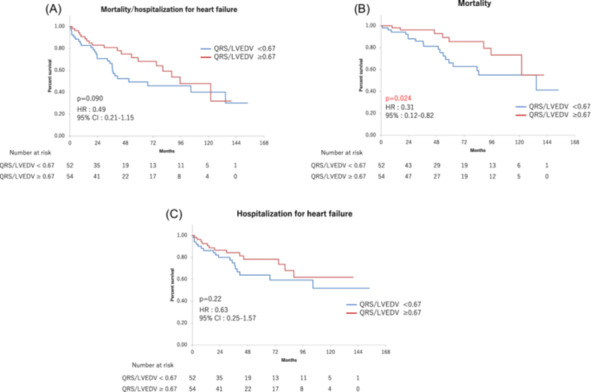
Kaplan−Meier curves for the probability of event‐free survival (QRSd/LVEDV ≥ 0.67 vs. QRSd/LVEDV < 0.67). Composite of all‐cause mortality and hospitalization for heart failure (A). All‐cause mortality (B) hospitalization for heart failure (C). LVEDV, left ventricular end‐diastolic volume; QRSd, QRS duration.

Although the two groups showed no significant differences in the incidence of the primary endpoint (35.1% vs. 51.9%, *p* = .49) (Figure 4A), patients with QRSd/LVEDV ≥ 0.67 showed better survival than patients with QRSd/LVEDV < 0.67 (14.8% vs. 34.6%, *p* = .0024) (Figure 4B). However, the groups showed no significant differences in the incidence of hospitalization for heart failure (24.1% vs. 34.6%, *p* = .63) (Figure 4C) or in the incidences of the secondary endpoints of appropriate defibrillator therapy (7.4% vs. 19.2%, *p* = .08; Figure SC).

## 4 DISCUSSION

The main findings of the present study were as follows: (1) The incidence of the composite primary endpoint of all‐cause death and hospitalization for heart failure was comparable between the groups showing LBBB with QRSd ≥120 milliseconds, non‐LBBB with QRSd ≥150 milliseconds, and non‐LBBB patients with QRSd of 120−149 milliseconds. (2) The incidence of the composite primary endpoint also showed no significant difference between patients with QRSd/LVEDV ≥ 0.67 and QRSd/LVEDV < 0.67. (3) However, patients with QRSd/LVEDV ≥ 0.67 showed better survival in this small‐body–size population.

### 4.1 Benefits of CRT according to QRSd and morphology

Previous randomized trials have shown that CRT improved the quality of life, including exercise tolerance, reduced LV volume, improved LV remodeling, and increased the LVEF in patients with NYHA class III/IV. In addition, the Comparison of Medical Therapy, Pacing, and Defibrillation in Heart Failure (COMPANION) study showed a significant reduction in all‐cause mortality or hospitalization for heart failure, and the CARE‐HF (Cardiac Resynchronization in Heart Failure) study showed a significant reduction in all‐cause mortality.^2^, ^3^ A meta‐analysis also showed that CRT reduced the total mortality by 26%.^8^

In the COMPANION and CARE‐HF studies, the mortality rate was significantly lower in patients whose QRS width was ≥148 and ≥160 milliseconds, respectively.^3^, ^8^ The effectiveness of CRT was not confirmed in the RethinQ (Resynchronization Therapy in Narrow QRS) study, which investigated patients with HF who showed dyssynchrony on echocardiography with NYHA class III, LVEF ≤ 35% and QRS width <130 milliseconds.^9^ Although the efficacy of CRT for patients with a relatively narrow QRS is still a matter of controversy, in the EchoCRT trial, HF patients with NYHA class III or IV, dyssynchrony on echocardiography and a QRS width of <130 milliseconds were randomly divided into the CRT‐on and CRT‐off groups, and their clinical outcomes were compared.^5^ The findings showed no significant difference between the two groups in all‐cause mortality or the incidence of hospitalization for HF (HR: 1.2, 95% CI: 0.92−1.57, *p* = .15).

Thus, the European Society of Cardiology (ESC) guidelines clearly state that CRT implantation is not recommended in patients with a QRSd of 120 milliseconds to define potential benefit; however, this will likely change. Furthermore, both the American College of Cardiology (ACC)/American Heart Association (AHA) and the ESC guidelines underline that evidence has become less clear when the QRS morphology is non‐LBBB and QRSd is between 130 and 150 milliseconds. In these situations, differences exist between the guidelines, with the ESC guideline providing a IIb recommendation for patients with non‐LBBB and QRSd of 130 to 149 milliseconds, and the ACC/AHA providing a class III recommendation.^10^

However, most of the previous RCTs and meta‐analyses demonstrating the effectiveness of CRT for CLBBB used registration criteria, including a QRS width of ≥120 milliseconds.^4^, ^7^, ^10^ Considering the characteristics of Japanese patients, who tend to be smaller than their Western counterparts, CRT can be considered useful for CLBBB patients with 120 milliseconds ≤QRS width <130 milliseconds. Thus, the lower limit of the QRS width should be set at 120 milliseconds in the CRT indication criteria of the JCS guideline. As shown in Table SA, the JCS, AHA/ACC, and ESC guidelines differ in terms of the indication level of CRT based on QRSd and morphology.

### 4.2 Clinical outcomes of CRT

The present study evaluated the incidence of cardiovascular events in patients with a small body size (BMI, 23.1) and advanced HF (NYHA class III/IV and LVEF > 35%) receiving CRT. The incidence of the composite of all‐cause death and hospitalization for HF was not significantly different among the groups showing LBBB with QRSd ≥120 milliseconds, non‐LBBB with QRSd ≥150 milliseconds, and non‐LBBB with QRSd of 120–149 milliseconds (9.34%, 12.03%, and 11.4% per year, respectively).

The recently published AdaptResponse trial enrolled an equal number of patients with NYHA class II and class III, whereas, in our study, 77% of the enrolled patients were with class III.^11^ This indicates that the registered patients in our study had more severe heart failure compared to those in the AdaptResponse trial, resulting in a higher proportion of patients meeting the primary endpoint.

There is an increasing recognition that QRSd may be prolonged not only by reduced myocardial conduction velocity but also by increased LV dimension acting to extend the “travel distance” of the propagating wavefront.^12^ This distinction is important because the former is the target for CRT, but the latter is influenced by HF remodeling, sex, and body size/height, thereby limiting the CRT response.^13^, ^14^ Interestingly, this calculation explained the inefficacy of CRT in some patients with LBBB and QRSd >150 milliseconds (i.e., those meeting class I CRT indications) and also explained why others (principally women) with a narrower QRSd (<150 milliseconds) were successfully treated by CRT. These observations raise the intriguing notion that some patients with QRSd <130 milliseconds, together with a smaller LV volume, may also benefit from CRT.

Furthermore, the septal flash, which is an echocardiographic index predicting the effectiveness of CRT, was recognized in >60% of female CLBBB patients with a small body surface area, even in patients with 120 milliseconds ≤QRS < 130 milliseconds.^15^ However, the conduction velocity and conduction path length might also play a role since the heart size in these patients is generally smaller. In patients eligible for CRT, however, using an identical QRSd cut‐off value (120 milliseconds) will lead to the selection of smaller hearts with poorer conduction. In other words, smaller hearts with a QRSd of >120 milliseconds will have a slower conduction velocity due to myocardial damage in comparison with larger hearts and might therefore be more amenable to successful treatment with CRT. The findings of this study suggest QRSd/LVEDV is a rather robust marker, underlining the possible importance of conduction velocity as a pivotal marker of CRT response. While previous studies have proposed predicting CRT responders by adjusting QRSd for height, our study found that height‐based interventions were ineffective.^16^

### 4.3 Study limitations

The present study had several limitations. First, this was a retrospective and single center study, and the number of included patients was limited and small. Second, diagnostic procedures were left to the discretion of physicians. Third, this study showed a higher prevalence of cardiac sarcoidosis, which can be attributed to the presence of a specialized outpatient clinic. Fourth, this study was not a randomized controlled trial, and the clinical outcomes of CRT were evaluated in a limited cohort of Japanese patients with small body size. Therefore, this study did not directly address the effectiveness of the QRSd/LVEDV ratio in patients with a CRTD. Further studies are needed to clarify whether CRTD implantation should be performed in patients with severe heart failure.

## 5 CONCLUSION

In conclusion, advanced HF patients with a higher QRSd/LVEDV ratio showed better survival in this small‐body–size population. These findings suggest that the risk is concentrated among those with a larger QRSd, and patients with a relatively smaller left ventricular size appeared to benefit from CRT. Thus, the QRSd/LVEDV might serve as a relatively simple index to improve patient selection for CRT in patients with mid‐wide QRSd.

## CONFLICT OF INTEREST STATEMENT

Dr. Yusuke Kondo received lecture fees from Daiichi‐Sankyo, Bayer, Abbott Medical Japan, Biotronik Japan, Boston Scientific, Japan Lifeline, and research funds from Daiichi‐Sankyo. Dr. Yoshio Kobayashi received lecture fees from Abbott Medical Japan, Bayer Japan, Bristol‐Myers Squibb, Boehringer Ingelheim, Daiichi‐Sankyo, and scholarship funds from Takeda Pharmaceutical, Abbott Medical Japan, Terumo, Otsuka Pharmaceutical, Boehringer Ingelheim, Astellas, Daiichi‐Sankyo, Win International, Japan Lifeline, and Nipro. The remaining authors declare no conflict of interest.

## Supporting information


**Figure A**. Kaplan–Meier curves for the probability of event‐free survival (class I vs. class IIa vs. class IIb). Appropriate ICD therapy. ICD, implantable cardioverter‐defibrillator. **Figure B**. Receiver‐operating characteristic (ROC) curve for predicting all‐cause death. The area under curve (AUC) was calculated to determine the best QRSd/LVEDV for predicting all‐cause death. ROC curve analysis confirmed that QRSd/LVEDV = 0.67 is best cut‐off point of predicting all‐cause mortality (AUC = 0.62). **Figure C**. Kaplan–Meier curves for the probability of event‐free survival (QRSd/LVEDV ≥ 0.67 vs, QRSd/LVEDV < 0.67 class). Appropriate ICD therapy. ICD, implantable cardioverter‐defibrillator.

**Table A**. Differences among the guidelines in terms of the indication level of CRT. JCS, the Japanese Circulation Society; ACC, American College of Cardiology; AHA, American Heart Association; CRT, cardiac resynchronisation therapy; HF, heart failure; LBBB, left bundle branch block; LVEF, left ventricular ejection fraction; OMT, optimal medical therapy.

## Data Availability

Raw data were generated at Chiba University. Derived data supporting the findings of this study are available from the corresponding author Yoshio Kobayashi on request.
