# Supplementary material for: Interaction of left ventricular size with the outcome of cardiac resynchronization therapy in Japanese patients
Source: Clin Cardiol. 2024 Apr 15;47(4):e24267. doi: 10.1002/clc.24267 (PMC11017297; doi:10.1002/clc.24267)
Supplement: Supplementary file 1 — Figure A. Kaplan–Meier curves for the probability of event‐free survival (class I vs. class IIa vs. class IIb). Appropriate ICD therapy. ICD, implantable cardioverter‐defibrillator. Figure B. Receiver‐operating characteristic (ROC) curve for predicting all‐cause death. The area under curve (AUC) was calculated to determine the best QRSd/LVEDV for predicting all‐cause death. ROC curve analysis confirmed that QRSd/LVEDV = 0.67 is best cut‐off point of predicting all‐cause mortality (AUC = 0.62). Figure C. Kaplan–Meier curves for the probability of event‐free survival (QRSd/LVEDV ≥ 0.67 vs, QRSd/LVEDV < 0.67 class). Appropriate ICD therapy. ICD, implantable cardioverter‐defibrillator. [file CLC-47-e24267-s002.pptx]

## Slide 1
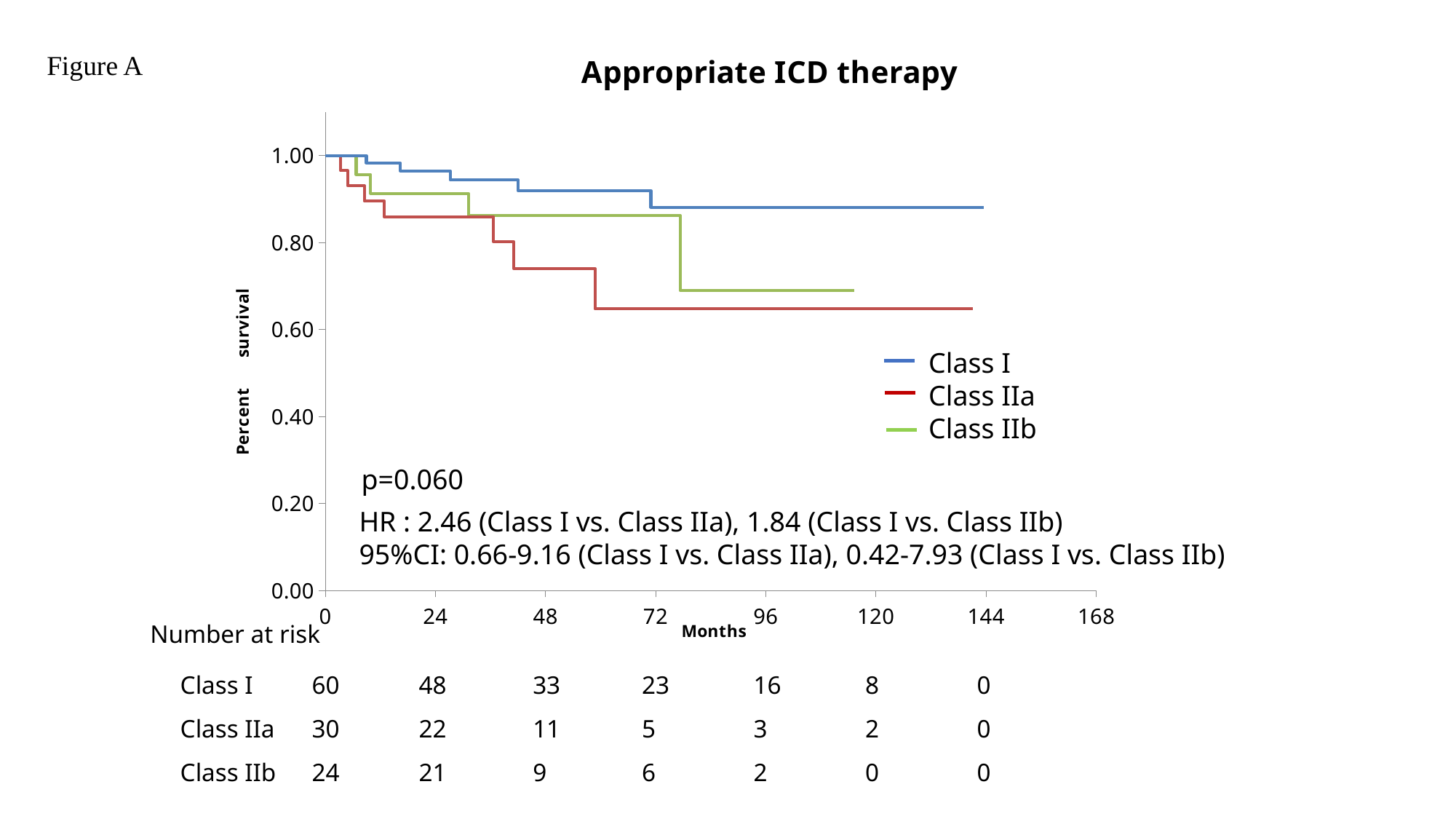

### Chart: Appropriate ICD therapy
| Category | Class I | Class IIa | Class IIb |
|---|---|---|---|Number at risk
Class I
Class IIa
Class IIb
60
30
24
48
22
21
33
11
9
23
5
6
16
3
2
8
2
0
0
0
0
Figure A
Class I
Class IIa
Class IIb
Class I
Class IIa
Class IIb
p=0.060
HR : 2.46 (Class I vs. Class IIa), 1.84 (Class I vs. Class IIb)
95%CI: 0.66-9.16 (Class I vs. Class IIa), 0.42-7.93 (Class I vs. Class IIb)

## Slide 2
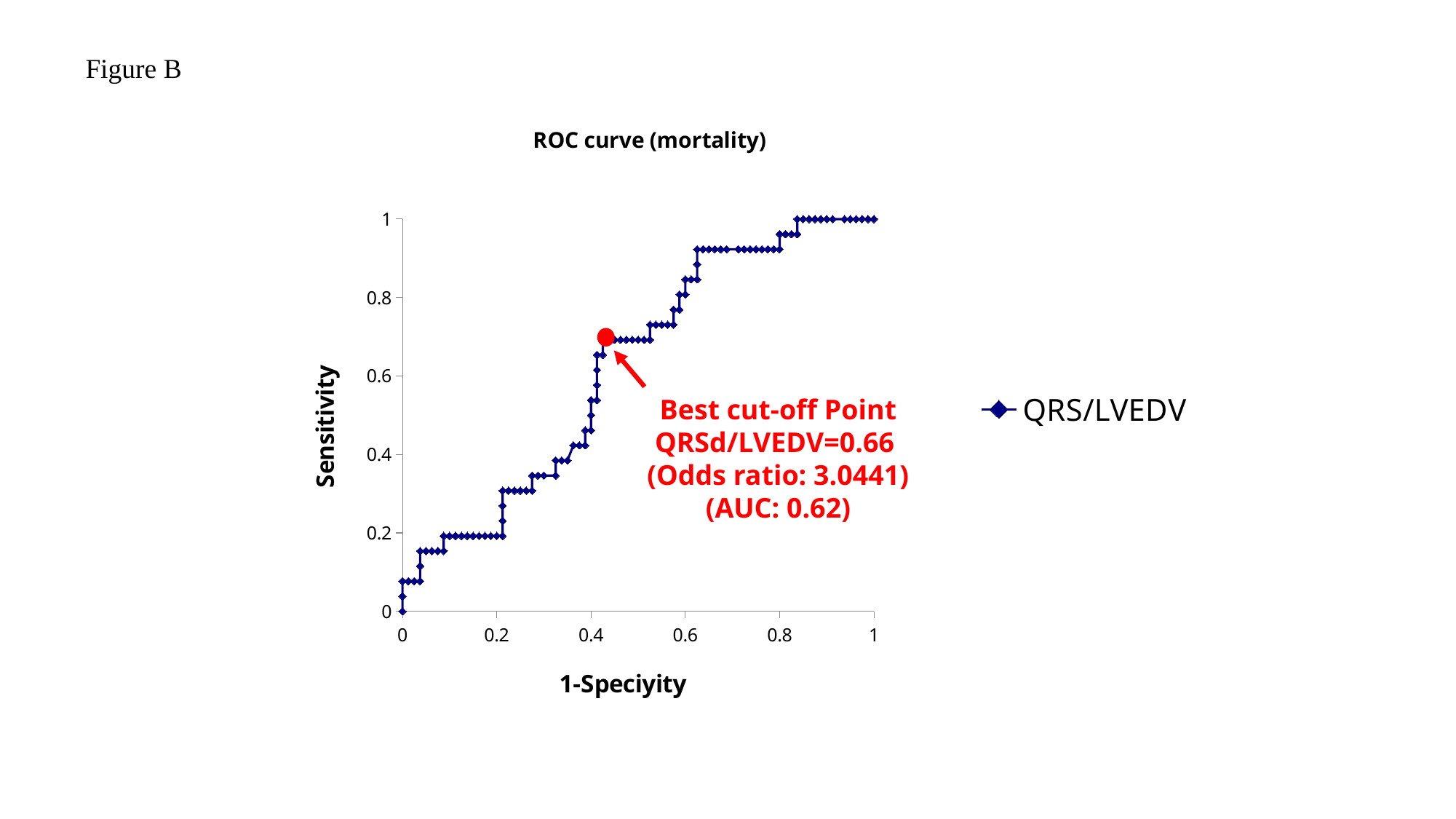

Figure B
### Chart: ROC curve (mortality)
| Category | QRS/LVEDV |
|---|---|
Best cut-off Point
QRSd/LVEDV=0.66
(Odds ratio: 3.0441)
(AUC: 0.62)

## Slide 3
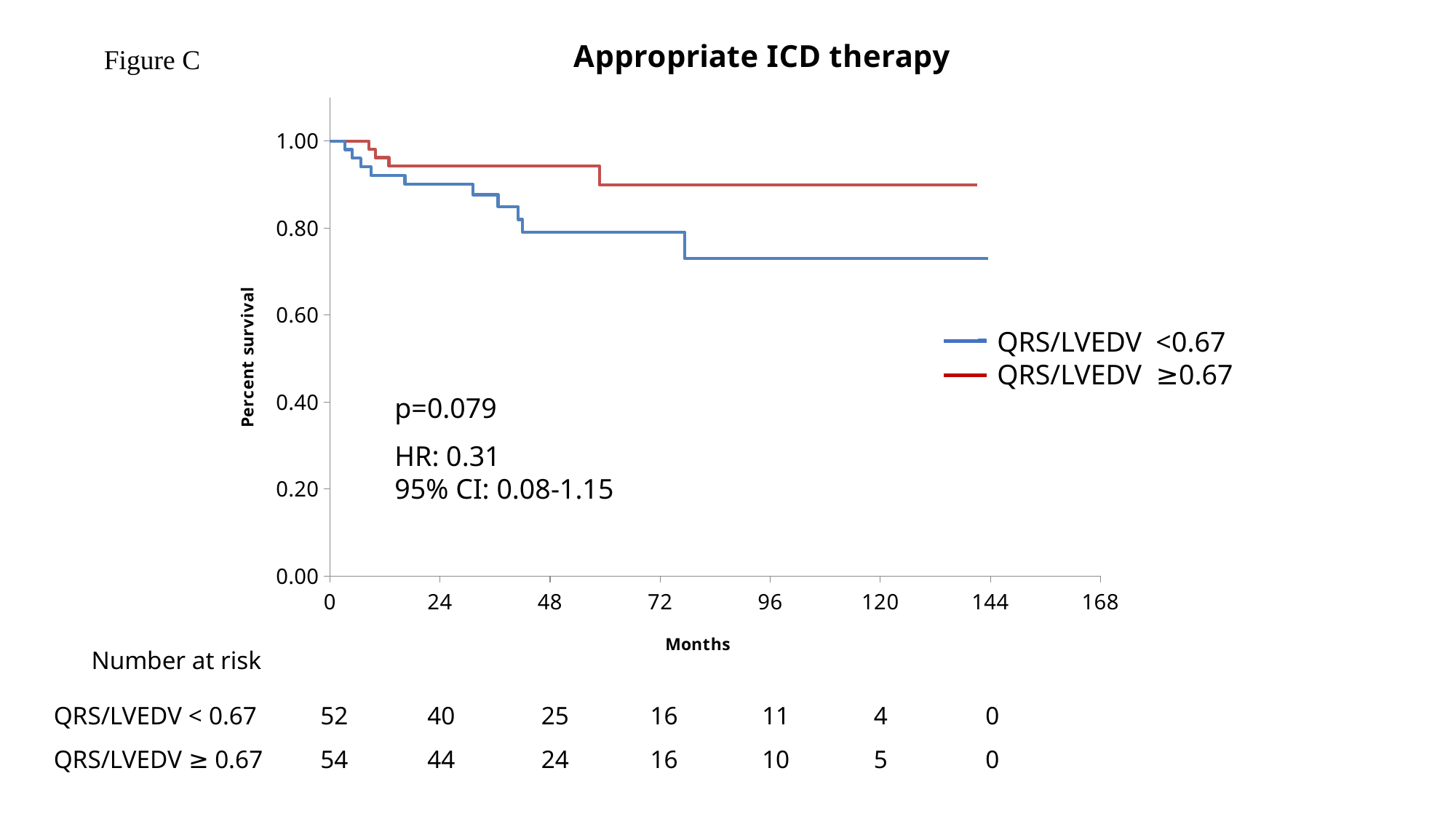

### Chart: Appropriate ICD therapy
| Category | QRS/LVEDV ≤0.66 | QRS/LVEDV >0.66 |
|---|---|---|Figure C
QRS/LVEDV <0.67
QRS/LVEDV ≥0.67
p=0.079
HR: 0.31
95% CI: 0.08-1.15
Number at risk
QRS/LVEDV < 0.67
QRS/LVEDV ≥ 0.67
52
54
40
44
25
24
16
16
11
10
4
5
0
0
