# Supplementary material for: Interaction of left ventricular size with the outcome of cardiac resynchronization therapy in Japanese patients
Source: Clin Cardiol. 2024 Apr 15;47(4):e24267. doi: 10.1002/clc.24267 (PMC11017297; doi:10.1002/clc.24267)
Supplement: Supplementary file 2 — Table A. Differences among the guidelines in terms of the indication level of CRT. JCS, the Japanese Circulation Society; ACC, American College of Cardiology; AHA, American Heart Association; CRT, cardiac resynchronisation therapy; HF, heart failure; LBBB, left bundle branch block; LVEF, left ventricular ejection fraction; OMT, optimal medical therapy. [file CLC-47-e24267-s001.pptx]

## Slide 1
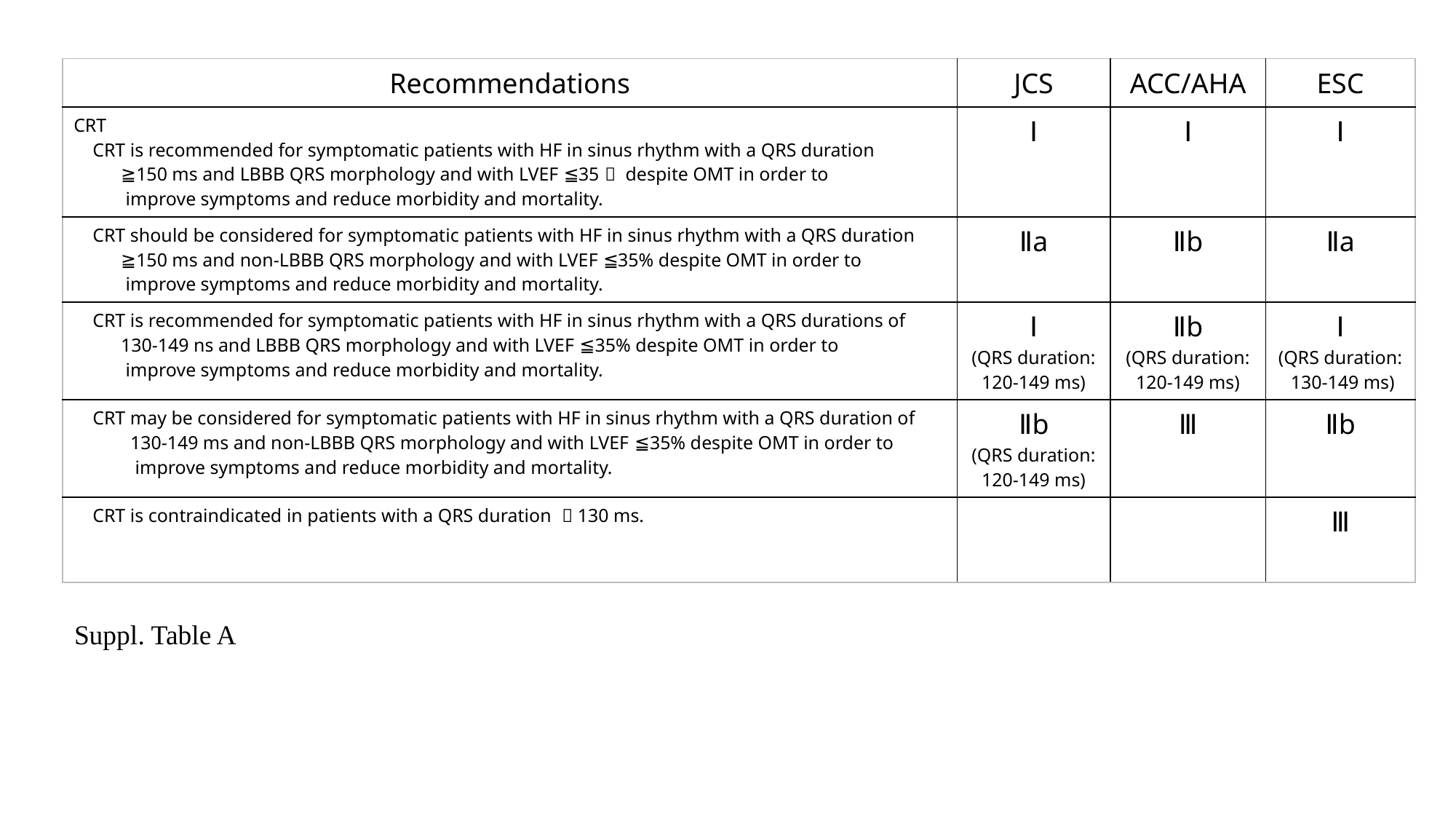

| Recommendations | JCS | ACC/AHA | ESC |
| --- | --- | --- | --- |
| CRT CRT is recommended for symptomatic patients with HF in sinus rhythm with a QRS duration ≧150 ms and LBBB QRS morphology and with LVEF ≦35％ despite OMT in order to improve symptoms and reduce morbidity and mortality. | Ⅰ | Ⅰ | Ⅰ |
| CRT should be considered for symptomatic patients with HF in sinus rhythm with a QRS duration ≧150 ms and non-LBBB QRS morphology and with LVEF ≦35% despite OMT in order to improve symptoms and reduce morbidity and mortality. | Ⅱa | Ⅱb | Ⅱa |
| CRT is recommended for symptomatic patients with HF in sinus rhythm with a QRS durations of 130-149 ns and LBBB QRS morphology and with LVEF ≦35% despite OMT in order to improve symptoms and reduce morbidity and mortality. | Ⅰ (QRS duration: 120-149 ms) | Ⅱb (QRS duration: 120-149 ms) | Ⅰ (QRS duration: 130-149 ms) |
| CRT may be considered for symptomatic patients with HF in sinus rhythm with a QRS duration of 130-149 ms and non-LBBB QRS morphology and with LVEF ≦35% despite OMT in order to improve symptoms and reduce morbidity and mortality. | Ⅱb (QRS duration: 120-149 ms) | Ⅲ | Ⅱb |
| CRT is contraindicated in patients with a QRS duration ＜130 ms. | | | Ⅲ |
Suppl. Table A
